# De novo transcriptome assembly data for sengon (*Falcataria moluccana*) trees displaying resistance and susceptibility to *boktor* stem borers (*Xystrocera festiva* Pascoe)

**DOI:** 10.1186/s13104-021-05675-9

**Published:** 2021-07-07

**Authors:** Ulfah J. Siregar, Aditya Nugroho, Hasyyati Shabrina, Fitri Indriani, Apriliya Damayanti, Deden D. Matra

**Affiliations:** 1 Department of Silviculture, Faculty of Forestry and Environment, IPB University (Bogor Agricultural University), Indonesia, Bogor, Indonesia; 2 SEAMEO BIOTROP, Bogor, Indonesia; 3 Tropical Silviculture Program, Department of Silviculture, Faculty of Forestry and Environment, IPB University (Bogor Agricultural University), Bogor, Indonesia; 4 Department of Agronomy and Horticulture, Faculty of Agriculture, IPB University (Bogor Agricultural University), Bogor, Indonesia

**Keywords:** *Falcataria moluccana*, Resistance, Transcriptome, *Xystroscera festiva*

## Abstract

**Objectives:**

Sengon (*Falcataria moluccana*) is a popular tree species in community plantation forests in Java, Indonesia due to its fast-growing and multipurpose characteristics. However, without effective control measures sengon plantations are vulnerable to *boktor* stem borer (*Xystrocera festiva*) infestation. Previous research found some *boktor*-resistant trees amid mostly susceptible individuals. Resistant trees have higher levels of enzyme inhibitory activity than susceptible ones. However, efforts to differentiate between the two accessions using microsatellite markers failed to provide satisfactory answers. This dataset was created to study differences in gene expressions between resistant and susceptible accessions, and to identify candidate genes involved in *boktor* resistance in sengon.

**Data description:**

RNA was extracted from fresh wood samples collected from two individual trees: one heavily infested with *boktor* larvae, and the other showing no signs of infestation. The sample trees grow in close proximity to each other within the same plantation. The RNA was sequenced using the BGISEQ-500 platform and produced 78.5 million raw reads. De novo transcriptome were assembled using Trinity and produced 96,164 contigs after filtering and clustering. This transcriptome data is important for understanding pest resistance mechanisms in sengon trees, serving as basis for an improvement program for resistance to *boktor* pest.

## Objective

Sengon (*Falcataria moluccana*) is a multipurpose legume tree, often utilized in reforestation programs and widely grown in community forest plantations in Indonesia, especially in Java. The fast-growing tree has high economic value, and can provide significant and rapid returns [1]. However, plantation productivity is being adversely affected by serious infestations of the larvae of a coleopteran stem borer known locally as *boktor* (*Xystrocera festiva*) [2]. The larvae feed on the cambium and outer parts of sapwood [3] causing deformities, wood quality degradation, and tree death. As there is no known effective method for their control [4], the selection of resistant tree lines is becoming an important option for establishing healthy stands. Previous research has shown that among mostly susceptible trees, some trees are resistant and have higher levels of enzyme inhibitory activity [5]. Efforts to differentiate between these two accessions using microsatellite markers have failed to provide satisfactory answers [6] as the mechanisms involved in tropical tree resistance to phytophagous pests remain largely unknown. Technological advances have allowed us to perform large-scale and rapid sequencing using next-generation sequencing (NGS) platforms to obtain genomic and transcriptomic data for perennial plants, especially trees, to accelerate tree improvement programs [7–9]. Therefore, this dataset was created to obtain differential expression information on candidate genes involved in *boktor* larvae resistance in sengon trees.

## Data description

Cambium samples were taken from two trees: resistant and susceptible trees in a community sengon plantation in Bogor, West Java, Indonesia (lat. -6.54416084, long. 106.7401301 DD). Trees showing no signs of infestation were considered resistant, while those heavily infested with *Xystroscera festiva* were deemed susceptible. A pair of trees, one heavily infested and the other showing no signs of infestation, were selected as samples. The sample trees had to be growing within the same cultivation plot in close proximity to each other in order to eliminate the possibility of environmental factors influencing the severity of pest infestation. Total RNA was extracted from 80 mg tissue samples using the established CTAB-pBIOZOL [10] method by following the manufacturer's instructions. The integrity and quantity of isolated-RNA were quantified by a NanoDrop ND-1000 spectrophotometer and Agilent 2100 Bioanalyzer. Before sequencing library construction, samples were treated with Ribo-zero rRNA remover [11] to remove the ribosomal RNA contaminant. RNA sequencing was performed using the BGISEQ-500 platform (BGI, Hong Kong).

The resulting raw reads (dataset 1) were then quality controlled using FastQC software [12] to ensure only high-quality data were used for further analysis. Clean reads were de novo assembled using Trinity v. 2.3.2 software [13, 14] and, due to high transcript redundancy, were processed further through filtering and clustering by using CAP3 [15], CD-HIT-EST [16] and Corset [17]. The clean reads were also mapped to reference genomes using Bowtie [18]. The assembled contigs (Data file 1), contained 96,164 contigs with an average length of 1,604.13 bp (Data file 2). Candidate proteins in coding sequences in all contigs were then extracted using TransDecoder v.5.5.0 [19] to produce Open Reading Frames (ORFs) predictions (Data file 3). The assembled contigs were also annotated using BLAST + [20] against the NCBI non-redundant protein (nr) (Data file 5), nucleotide sequence (nt) (Data file 6), and SwissProt protein sequence databases (Data file 7) and TrEMBL from UniProt (Data file 8), with an E-value cut-off − 10 [21].

Transcriptome reference statistics were then analyzed using Blast2GO in OmicsBox [22] to produce distribution data on species blasted, top-hit species blasted, E-value, and sequence similarity (Data file 9). Gene ontology and KEGG pathway analyses were performed using contigs annotated with the Swiss-Prot database (Data file 10), locating 31 cellular components, 38 molecular functions, 60 biological processes (Data file 11), and 148 pathways (Data file 12). Microsatellite regions (Data file 13) in contigs were found using MISA [23] with minimum repeats: 10 for one base; 6 for two bases; and 5 for 3, 4, 5 and 6 bases; and the maximum interruptions allowed between two or more microsatellite sites were 100 bases. The number of contigs containing microsatellite regions was 37,956 contigs with 57,487 microsatellite sites identified (Data file 14).

## Limitations

The infested sample was collected from wood around holes made by *boktor* larvae at 1.5 m height and not at the initial stage of the infestation. The infestation occurred in an uncontrolled manner since it was on open land, but the two trees sampled were only two meters apart. The number of samples sequenced in this study was limited to one sample each for two conditions due to the insufficient RNA quality of other samples for further processing.

## Data Availability

The data described in this data note can be accessed from the DNA Data Bank of Japan (DDBJ) with accession number DRP007012, and Figshare https://doi.org/10.6084/m9.figshare.14058458.v1. Please see Table

**Table 1:** Overview of data files/dataset

Label	Name of data	File types (extension)	Data repository and accession number
Dataset 1	Raw RNA-seq reads	Fastq files (.fastq)	DNA Data Bank of Japan (DDBJ) accession number DRP007012 https://trace.ddbj.nig.ac.jp/DRASearch/study?acc=DRP007012 [24]
Data file 1	Transcriptome assembly contigs	Fasta file (.fasta)	Figshare https://doi.org/10.6084/m9.figshare.14058458.v1 [25]
Data file 2	Summary for alignment of clean reads to reference transcriptome	Document file (.docx)	Figshare https://doi.org/10.6084/m9.figshare.14058458.v1
Data file 3	Open reading frames (ORFs) prediction	Fasta file (.fasta)	Figshare https://doi.org/10.6084/m9.figshare.14058458.v1
Data file 4	Open reading frames (ORFs) summary	Document file (.docx)	Figshare https://doi.org/10.6084/m9.figshare.14058458.v1
Data file 5	Functional annotation from non-redundant (nr) protein NCBI	BLAST output in XML/-outfmt 5 option	Figshare https://doi.org/10.6084/m9.figshare.14058458.v1
Data file 6	Functional annotation from non-redundant nucleotide (nt) NCBI	Text file (.txt)	Figshare https://doi.org/10.6084/m9.figshare.14058458.v1 [23]
Data file 7	Functional annotation from Swiss-Prot	Text file (.txt)	Figshare https://doi.org/10.6084/m9.figshare.14058458.v1
Data file 8	Functional annotation from TrEMBL UniProt	Text file (.txt)	Figshare https://doi.org/10.6084/m9.figshare.14058458.v1
Data file 9	Statistics related to contig length distribution and the Blast results: e-value distribution, contig similarity distribution, top-hit species distribution	Compressed PNG files (.rar)	Figshare https://doi.org/10.6084/m9.figshare.14058458.v1
Data file 10	Gene Ontology and KEGG analysis	Blas2GO file (.b2g)	Figshare https://doi.org/10.6084/m9.figshare.14058458.v1
Data file 11	Summary of gene ontology	Compressed text files (.rar)	Figshare https://doi.org/10.6084/m9.figshare.14058458.v1
Data file 12	KEGG Pathway Summary	Text file (.txt)	Figshare https://doi.org/10.6084/m9.figshare.14058458.v1
Data file 13	Results of microsatellite region finding	MISA file (.misa)	Figshare https://doi.org/10.6084/m9.figshare.14058458.v1
Data file 14	Statistics of microsatellite regions	STATISTICS file (.statistics)	Figshare https://doi.org/10.6084/m9.figshare.14058458.v1

^1^ and the list of references [24, 25] for details and links to the data. Overview of data files/dataset

## Abbreviations

CTAB: Cetyltrimethylammonium bromide
RNA: Ribonucleic acid
RNA-seq: RNA sequencing
nr: Non-redundant protein
nt: Nucleotide sequences
TrEMBL: Translated European Molecular Biology Laboratory
KEGG: Kyoto Encyclopedia of Genes and Genomes
